# Impact of Type and Screen Method on Turnaround Time and Man-Hour Utilization Compared to Conventional Coomb’s Crossmatch: A Cross-Sectional Analytical Study

**DOI:** 10.7759/cureus.69564

**Published:** 2024-09-16

**Authors:** Arthi R, Soundharya V, Suresh Kumar I, Hari Haran A, Sahayaraj James

**Affiliations:** 1 Transfusion Medicine, Saveetha Medical College and Hospital, Saveetha Institute of Medical and Technical Sciences, Chennai, IND

**Keywords:** ahg crossmatch, blood bank, man-hour utilisation, turnaround time, type and screen

## Abstract

Background

Blood component transfusion is vital for various medical conditions, requiring thorough pretransfusion testing to ensure safety and compatibility. The Type and Screen (T and S) method allows for efficient detection of clinically significant antibodies while reducing unnecessary crossmatching. This study evaluates T and S’s impact on turnaround time and man-hour utilization compared to conventional crossmatching.

Methodology

The study included 835 elective crossmatch requests. Blood grouping, antibody screening, and antihuman globulin (AHG) crossmatching were performed using column agglutination technology, while immediate saline crossmatching was done by tube technique. Turnaround time (TAT), man-hour utilization, and saved rates were calculated for both the T and S protocol and routine AHG crossmatch.

Results

In this study, 835 elective blood samples underwent antibody screening and immediate saline phase crossmatching, with validation by AHG phase crossmatch. The T and S protocol significantly reduced TAT for both first (51.24 vs. 71.56 minutes) and subsequent transfusions (17.47 vs. 39.67 minutes) compared to AHG crossmatching, with reductions being statistically significant (p < 0.0001). T and S also saved 279.28 man-hours (11.6 man-days), equating to 0.33 man-hours saved per sample.

Conclusion

Our study shows that the T and S protocol significantly enhances blood bank efficiency by reducing TAT and man-hour utilization compared to conventional AHG crossmatching. This improvement not only optimizes manpower but also makes the process more cost-effective.

## Introduction

Blood component transfusion is a crucial therapeutic intervention for various diseases, surgeries, and medical conditions. Prior to administering blood components, pretransfusion testing plays a crucial role in ensuring the safety and compatibility of the transfusion. ABO grouping, Rh typing, auto- and alloantibody screening, antibody identification, and compatibility testing are all included in pre-transfusion testing [1]. Compatibility or major cross-match testing is often conducted in two stages. Immediate spin (IS) saline phase is conducted at room temperature (20-25°C) to rule out IgM antibodies. This phase lasts for approximately ten to fifteen minutes. Antihuman globulin (AHG), often known as the Coombs phase, is used to identify non-agglutinating incomplete IgG antibodies [2]. This process takes thirty to sixty minutes.

Antibody screening (AS) became part of pretransfusion testing in the early 1960s alongside the AHG phase crossmatch, primarily targeting clinically significant non-ABO IgG antibodies. However, the necessity of AHG phase crossmatching was questioned when AS results were negative, leading to the adoption of the Type and Screen (T and S) approach [3]. T and S procedure is one of the effective approaches in pre-transfusion testing in which antibody screen negative recipients are transfused with immediate saline phase compatible packed red blood cell (PRBC) unit. ‘Typing and Screening’ includes ‘typing the patient’s red cells for ABO, Rh blood group, and screening patient’s serum for the presence of unexpected antibodies in the presence of reagent red cells in an AHG phase’ [4].

T and S offers significant advantages in clinical practice, patient safety, blood bank operations, and adherence to hospital policies [5]. This method allows for the detection and identification of clinically significant antibodies and facilitates the search for antigen-negative blood units. T and S is particularly beneficial in situations where the likelihood of transfusion is low, as it prevents unnecessary crossmatching, thereby reducing the workload on blood banks. Additionally, crossmatched blood is reserved and remains inaccessible for other patients in need for 24-48 hours as per hospital policy. The routine issuance of blood using the AHG technique takes approximately 90 minutes, whereas the T and S method enables blood to be issued within 10-15 minutes [5].

The majority of research showed that individuals receiving blood transfusions utilizing the T and S technique did not experience delayed hemolytic transfusion reactions. Despite the fact that the AHG crossmatch that followed suggested that some transfused red blood units were incompatible, none of the patients had any clinically or serologically detectable hemolysis [6]. Therefore, pretransfusion testing can be performed without endangering patients by omitting the AHG crossmatch. Studies have also shown that the T and S method is safe for patients who need large amounts of blood transfusions repeatedly [7].

The impact of the Type and Screen method on turnaround time (TAT) and manpower, compared to the conventional crossmatch method, has been scarcely studied in our population. This study aimed to evaluate the effects of the conventional antiglobulin crossmatch procedure versus the Type and Screen method on both turnaround time and the man-hours utilized.

## Materials and methods

This was a cross-sectional analytical study done in the Department of Transfusion Medicine, Saveetha Medical College and Hospital. The study was done over 4 months from January 2024 to April 2024. The study included all elective crossmatch requests that were received at the blood centre. Samples of patients with a previous history of transfusion or pregnancy were also included in the study. Patient samples with known allo-antibody were excluded from the study.

In the study done by Chaudhary et al. [4], the specificity of immediate spin cross-matching to predict compatibility was 99.25%. With an expected specificity of 99.25%, an expected prevalence of compatibility of 98.0%, and a 95% confidence interval with 5% absolute precision the sample size was calculated as 823. By consecutive sampling, we have processed a total of 835 samples in the study period of 4 months. This included 640 samples of patients undergoing transfusion for the first time and 195 samples of patients who were having subsequent transfusions.

Patient and donor blood grouping, antibody screening and AHG crossmatch were done by column agglutination technology (CAT). Immediate saline crossmatch was done by tube technique. Antibody screening in all samples was done using a commercial 3-cell reagent panel. All samples were validated by AHG crossmatch before the issue.

**Figure 1:**
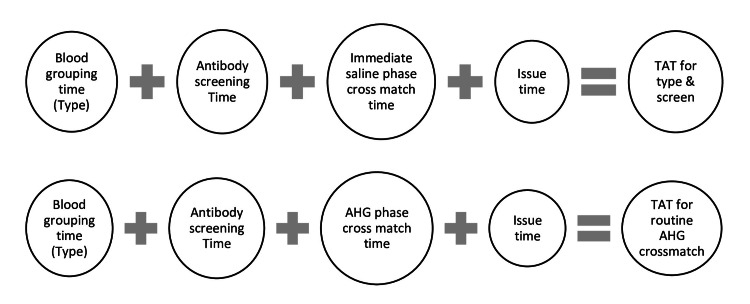
TAT calculation for first transfusion in T and S protocol and routine AHG crossmatch TAT: turnaround time; T and S: Type and Screen; AHG: antihuman globulin

The turnaround time (TAT) during processing study samples for the first time for Type and Screen with AHG validation taken simultaneously, was calculated in minutes as shown in Figure 1.

**Figure 2:**
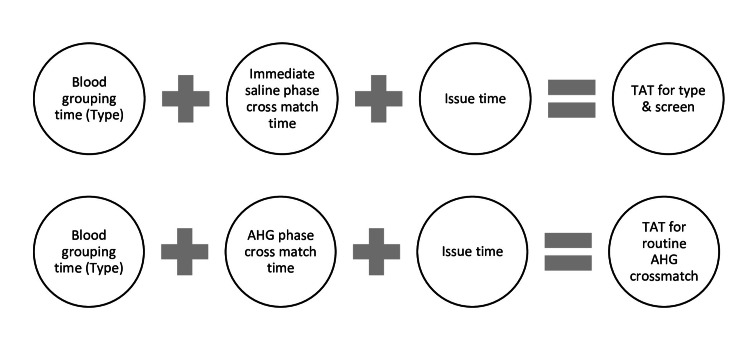
TAT calculation for subsequent transfusion in T and S protocol and routine AHG crossmatch TAT: turnaround time; T and S: Type and Screen; AHG: antihuman globulin

The TAT during processing study samples for subsequent transfusions for T and S with AHG validation, was calculated simultaneously in minutes as shown in Figure 2.

Man-hour utilization and saved rate were calculated by multiplying the total number of units cross-matched with the total mean TAT taken for immediate saline and AHG phase cross-match protocol respectively.

All statistical analyses were done by using SPSS version 21 (IBM Corp., Armonk, USA). The study included only cross-matching samples that were received in the blood bank. Ethical clearance was obtained from the Institutional Ethical Committee with registration number 097/12/2023/PG/SRB/SMCH. 

## Results

In our study, 835 elective blood samples were subjected to antibody screening by reagent 3 red cell screening panel, and antibody screen negative blood samples were subjected to immediate saline phase cross match. The same samples were subjected to AHG phase crossmatch for validation.

The mean age of our study population was 42 ± 7.4SD years. Among the 835 study population, 433(51.8%) were males, and 402 (48.1%) were females. Among our study population, majority of patients belong to O blood group which comprises 386 (46.2%), followed by B blood group which comprises 235 (28.1%) and A blood group 214 (25.6%). The majority of blood requests around 63.7% (N= 532) were indicated for surgery as a part of the request by the treating surgeon, followed by anemia correction which contributed around 12.4% (N= 104). Out of 835 patients, no case was found to be positive for antibody screening.

Turnaround Time (TAT) for routine AHG phase cross match and Type and Screen protocol for first transfusion (N= 640) among study samples are shown in Table 1 and Table 2, respectively. Hence, in our study, the mean total turn around time for routine AHG phase crossmatch was found to be 71.56 ± 5.78 SD minutes. The mean total turn around time with type and screen protocol for first transfusion was found to be 51.24 ± 3.6 SD minutes. Hence, we can observe a considerable reduction in TAT with the Type and Screen protocol.

**Table 1:** TAT for type and screen protocol for first transfusion among study samples (N = 640) TAT: turnaround time

Mean blood grouping time (in minutes)	Mean antibody screening time (in minutes)	Mean immediate saline phase crossmatching time (in minutes)	Mean issue time (in minutes)	Mean total turn around time (in minutes)
5.64 ± 0.32 SD	32.18 ± 2.36 SD	11.30 ± 0.62 SD	2.12 ± 0.3 SD	51.24 ± 3.6 SD

**Table 2:** TAT for routine AHG phase cross match with first transfusion among study samples (N = 640) TAT: turnaround time; AHG: antihuman globulin

Mean blood grouping time (in minutes)	Mean antibody screening time (in minutes)	Mean AHG phase crossmatching time (in minutes)	Mean issue time (in minutes)	Mean total turn around time (in minutes)
5.64 ± 0.32 SD	32.18 ± 2.36 SD	31.62 ± 2.8 SD	2.12 ± 0.3 SD	71.56 ± 5.78 SD

**Figure 3:**
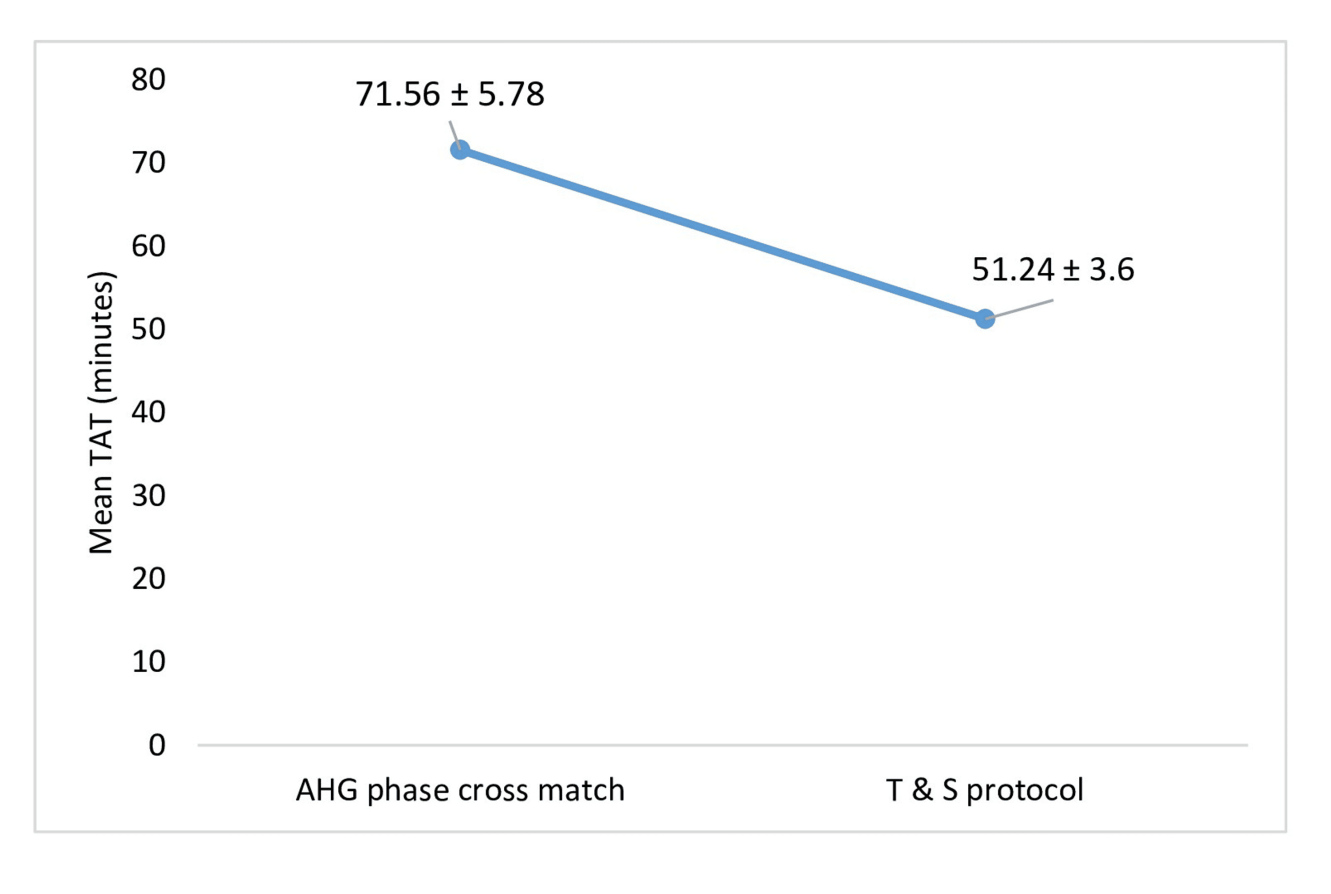
Mean TAT for first transfusion in T and S protocol and routine AHG crossmatch TAT: turnaround time; T and S: Type and Screen; AHG: antihuman globulin

The reduction in mean total TAT for first transfusion from 71.56 ± 5.78 SD minutes with AHG phase crossmatch to 51.24 ± 3.6 SD minutes with Type and Screen protocol was statistically significant (p < 0.0001) as shown in Figure 3.

TAT for subsequent transfusions with routine AHG phase cross match and with type and screen protocol among study samples (N = 195) are shown in Table 3 and Table 4, respectively. The TAT for subsequent transfusion with routine AHG phase cross match was found to be 39.67 ± 3.3 SD minutes. Hence in our study mean total turn around time for subsequent transfusion has drastically reduced from 39.31 ± 3.3 SD minutes with AHG phase cross match to 17.47 ± 1.2 SD minutes with Type and screen protocol.

**Table 3:** TAT for subsequent transfusions with routine AHG phase cross match (N= 195) TAT: turnaround time; AHG: antihuman globulin

Mean blood grouping time (in minutes)	Mean AHG phase crossmatching time (in minutes)	Mean issue time (in minutes)	Mean total turn around time (in minutes)
5.31 ± 0.22 SD	32.18 ± 2.8 SD	2.18 ± 0.28 SD	39.67 ± 3.3 SD

**Table 4:** TAT for subsequent transfusion with Type and Screen protocol (N = 195) TAT: turnaround time

Mean blood grouping time (in minutes)	Mean immediate saline phase crossmatching time (in minutes)	Mean issue time (in minutes)	Mean total turn around time (in minutes)
5.31 ± 0.22 SD	9.98 ± 0.7 SD	2.18 ± 0.28 SD	17.47 ± 1.2 SD

**Figure 4:**
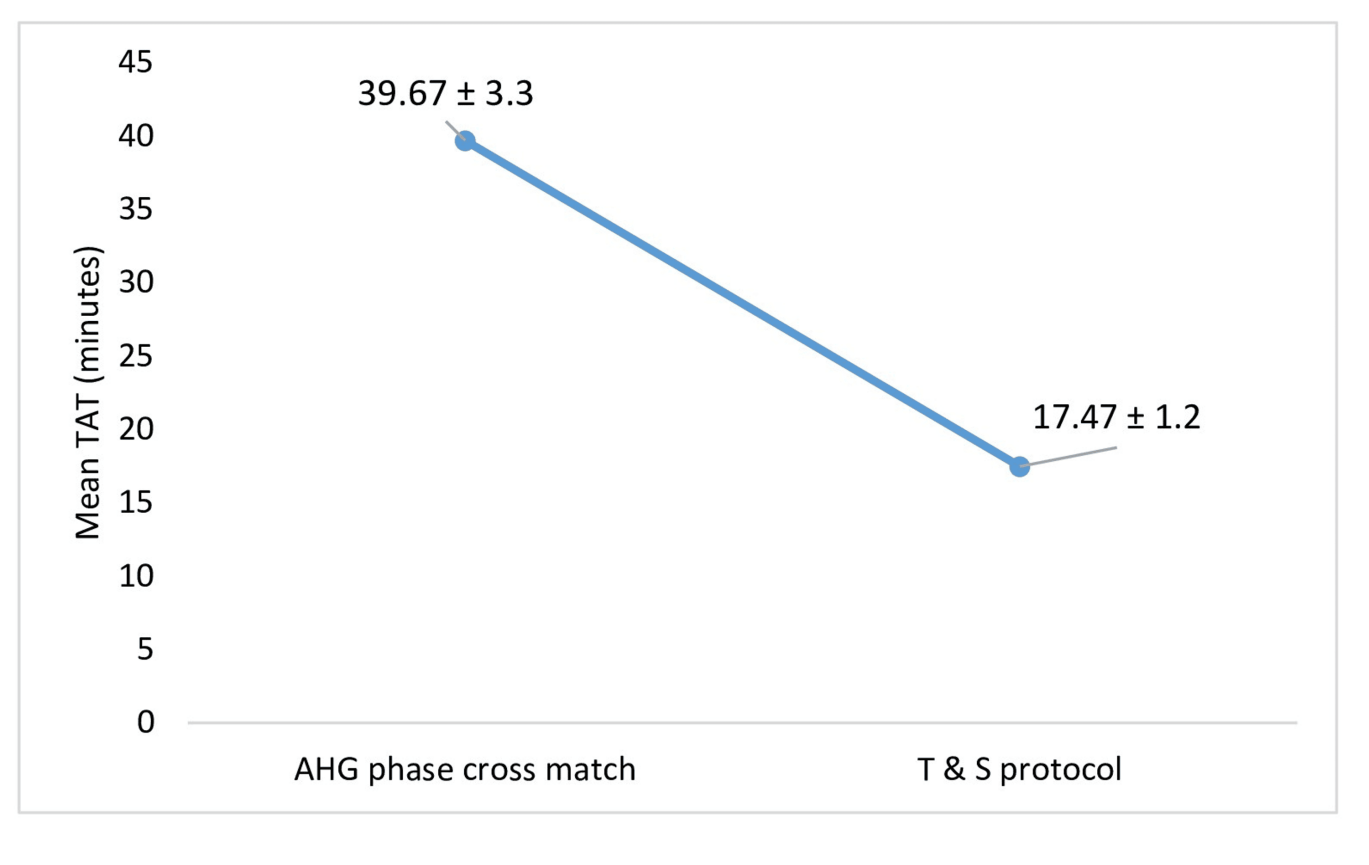
Mean TAT for subsequent transfusion in T and S protocol and routine AHG crossmatch TAT: turnaround time; T and S: Type and Screen; AHG: antihuman globulin

The decrease in mean total TAT for subsequent transfusions from 39.67 ± 3.3 SD minutes with AHG phase cross match to 17.47 ± 1.2 SD minutes with Type and Screen protocol was statistically significant (p < 0.0001) as shown in Figure 4.

**Figure 5:**
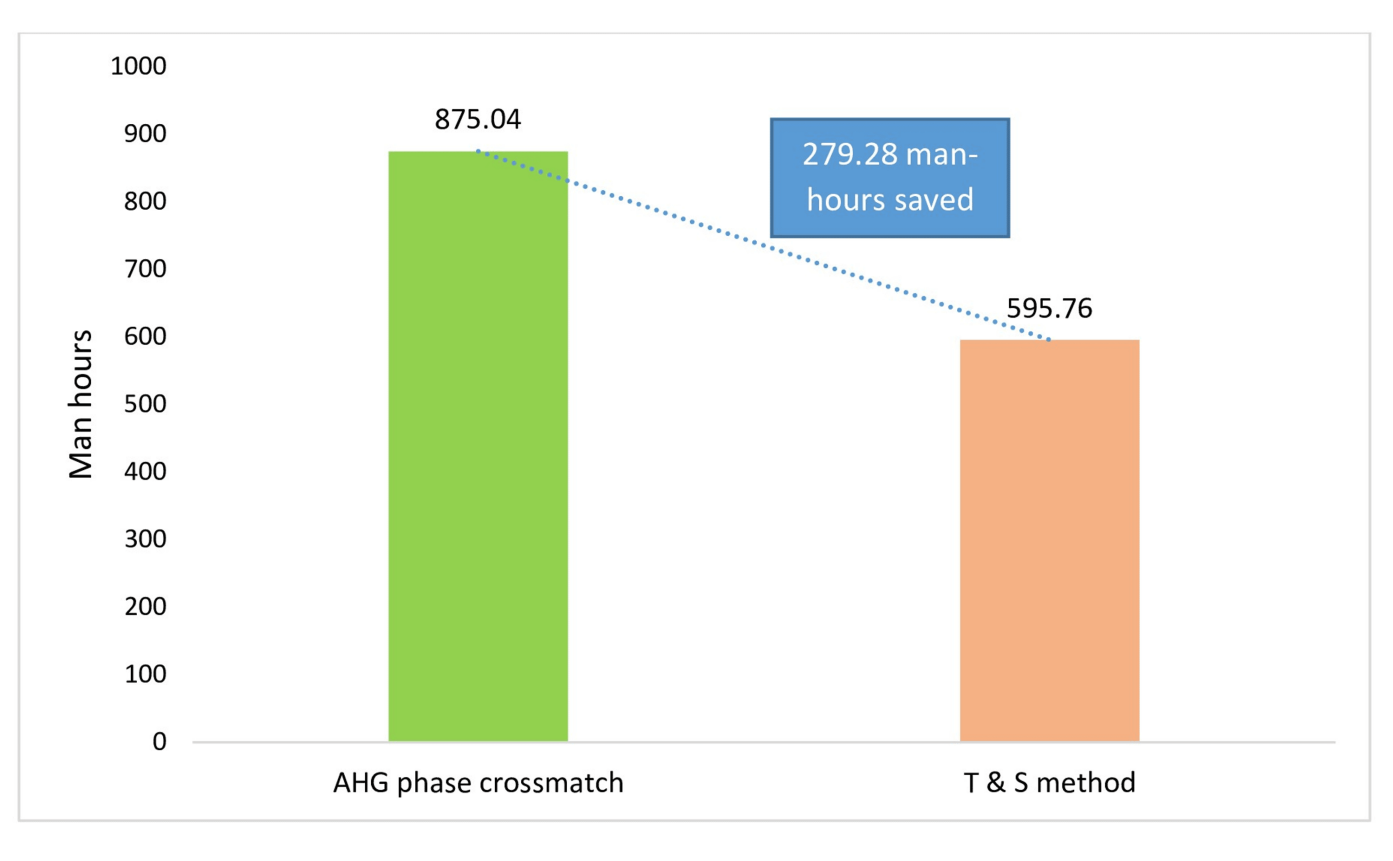
Man-hour utilization and saved rates in T and S protocol and routine AHG crossmatch T and S: Type and Screen; AHG: antihuman globulin

Man-Hour Utilization and Saved Rate were calculated by multiplying the total number of units cross matched with the total mean TAT taken for immediate saline and AHG phase cross match protocol respectively. For our study samples, it was 875.04 man-hours for AHG phase crossmatch. For Type and Screen protocol, when calculated similarly we found it to be 595.76 man-hours. The total man-hours saved in our study is 279.28 man-hours, i.e., around 11.6 man-days. This corresponds to 0.33 man-hours saved per sample. Thus, there is a significant reduction in man-hours utilization with type and screen protocol as shown in Figure 5. The man-hours saved can be directed for other effective and useful tests in blood banks.

## Discussion

In most Indian hospitals blood units are fully cross-matched and reserved for patients, even though the likelihood of many of them needing a transfusion is low. Blood units that are not issued to patients within the allotted time frame are considered "unreserved" and are returned to the main inventory. As a result, the chance of blood units expiring increases due to repeated reservations and cancellations of reserved units. In these circumstances, a T and S policy would be the best option.

Several studies have evaluated the T and S protocol against conventional AHG crossmatch. A study by Chaudhary et al showed that the T and S procedure gave a safety of 91.6% [4]. Tiwari et al studied 2402 patients with negative AS [8]. 99.7% of red cell units that were compatible with IS were also compatible on post transfusion AHG crossmatch. One patient who received two IS crossmatch compatible units was found to have anti-P1 alloantibody; however, both units later proved to be incompatible on AHG crossmatch. Kumar et al. demonstrated that the AHG phase crossmatch and the T and S protocol were in perfect agreement. The T and S protocol was implemented, resulting in a reduction of the crossmatch transfusion ratio from 2.1 to 1.5 [9]. Kuriyan et al. concluded that it is safe to eliminate the crossmatch from patients who do not have red cell antibodies as long as an error detection system is in place [10].

The T and S method has certain drawbacks, such as the possibility of missing antibodies against antigens that are not present on the screening cell panel, which is particularly relevant for the South Asian population, as most cell lines are derived from the Caucasian population [11]. However, in a patient with a negative AS (false negative), the probability of missing a clinically significant red cell antibody is 1-4/10,000 [12]. Additionally, antibodies may go undetected if the screening cell panel exhibits only a weak or heterogeneous expression of the corresponding antigen. Furthermore, the T and S method requires specific infrastructure and technical expertise, which may not be accessible in all centers across India.

In our study we analysed the TAT and man-hour utilization for routine AHG crossmatch and T and S protocol. TAT serves as a key quality indicator for laboratory services [13]. Our study revealed a significant reduction in TAT, dropping from 71.56 minutes to 51.24 minutes when using the T and S protocol for the first transfusion, with the difference being statistically significant (p<0.001). For subsequent transfusions, the TAT decreased even further, from 39.67 minutes to 17.47 minutes with the T and S protocol. These findings align with those of Aggarwal et al. [5], who observed a reduction in TAT from 79.71 minutes to 65.61 minutes after implementing the T and S protocol. Similarly, studies by Alavi-Moghaddam et al. [14] and Chow [15] also demonstrated a significant decrease in TAT following the adoption of the T and S method. Numerous studies have confirmed a significant reduction in TAT; however, the degree of reduction varies among studies due to differing definitions of TAT in each study.

Our study found a significant reduction in man-hours utilized with the implementation of the T and S protocol. The time saved can be reallocated to other essential and valuable tasks in the blood bank. The T and S protocol decreases the number of units that need to be crossmatched, thereby reducing the time required for crossmatching and resulting in considerable savings in man-hours compared to the AHG phase crossmatch. In the study by Aggarwal et al [5] with a sample size of 24,724 there was a reduction in man utilization hours from 32,466.1 hours to 27,039.2 hours with type and screen protocol, the man-hours saved is 5427 man-hours which works out to 226 man-days. This accounts for 0.32 man-hours saved per sample. As per our study, we can save 0.33 man-hours per sample which is comparable with the Aggarwal et al study [5].

In our study, the Type and Screen protocol was fully validated by the AHG crossmatch, with no incompatibilities detected. This indicates that the T and S protocol demonstrates a 100% safety value among elective patients. However multicentric studies with a larger sample, including urgent crossmatch requests have to be conducted to further validate the T and S protocol.

The limitations of this study include its single-center design, which may limit the generalizability of the findings to other settings. The application of the T and S protocol was only evaluated for elective blood transfusion requests in this study; its effectiveness in urgent and emergency situations still needs to be investigated. Also the potential for undetected antibodies that might emerge with larger or more diverse patient populations was not studied. 

## Conclusions

Our study demonstrates that the Type and Screen protocol offers substantial improvements in blood bank efficiency. By significantly reducing both turnaround time and man-hour utilization compared to the conventional AHG crossmatch method, the T and S protocol not only expedites the transfusion process but also frees up valuable resources.

The man-hours saved allows blood bank staff to redirect their efforts toward other critical tasks. T and S protocol also saves the cost of reagents, thus enhancing overall operational efficiency. These benefits highlight the T and S protocol as a more effective and economical approach to managing blood transfusion services.

## References

[1] Sarah K, Harm NM (2017). Pretransfusion testing and storage, monitoring, processing, distribution and inventory management of blood components. Technical Manual.

[2] Trudeau LR, Judd WJ, Butch SH, Oberman HA (1983). Is a room-temperature crossmatch necessary for the detection of ABO errors?. Transfusion.

[3] GR-GR M (1964). Routine compatibility testing: standards of the aabb as applied to compatibility tests. Transfusion.

[4] Chaudhary R, Agarwal N (2011). Safety of type and screen method compared to conventional antiglobulin crossmatch procedures for compatibility testing in Indian setting. Asian J Transfus Sci.

[5] Aggarwal G, Tiwari AK, Arora D, Dara RC, Acharya DP, Bhardwaj G, Sharma J (2018). Advantages of type and screen policy: Perspective from a developing country!. Asian J Transfus Sci.

[6] Heddle NM, O'Hoski P, Singer J, McBride JA, Ali MA, Kelton JG (1992). A prospective study to determine the safety of omitting the antiglobulin crossmatch from pretransfusion testing. Br J Haematol.

[7] Oberman HA, Barnes BA, Friedman BA (1978). The risk of abbreviating the major crossmatch in urgent or massive transfusion. Transfusion.

[8] Tiwari AK, Aggarwal G, Dara RC, Arora D, Gupta GK, Raina V (2017). First Indian study to establish safety of immediate-spin crossmatch for red blood cell transfusion in antibody screen-negative recipients. Asian J Transfus Sci.

[9] Kumar IS, Kulkarni RG, Sahoo D, Basavarajegowda A Implementing type and screen method replacing conventional antiglobulin crossmatch procedure for compatibility testing in elective protocol in a tertiary care hospital. Asian J Transfus Sci.

[10] Kuriyan M, Fox E (2000). Pretransfusion testing without serologic crossmatch: approaches to ensure patient safety. Vox Sang.

[11] Setia R, Sachdeva P, Arora S, Handoo A, Kapoor M (2017). Making type and screen policy an essential component of pretransfusion testing: need of the hour in India. Glob J Transfus Med AATM.

[12] Mintz PD, Haines AL, Sullivan MF (1982). Incompatible crossmatch following nonreactive antibody detection test. Frequency and cause. Transfusion.

[13] Dey B, Bharti JN, Chakraborty M (2013). Laboratory turnaround time. Int J Health Sci Res.

[14] Alavi-Moghaddam M, Bardeh M, Alimohammadi H, Emami H, Hosseini-Zijoud SM (2014). Blood transfusion practice before and after implementation of type and screen protocol in emergency department of a university affiliated hospital in Iran. Emerg Med Int.

[15] Chow EYD (1999). The impact of the type and screen test policy on hospital transfusion practice. Hong Kong Med J.

